# Public health round-up

**DOI:** 10.2471/BLT.22.011222

**Published:** 2022-12-01

**Crisis in the Horn of Africa**

People waiting for water troughs to fill at Hula Hula Springs in Marsabit County, Kenya, where communities are suffering from the worst drought in 40 years. Kenya is one of seven countries in the Horn of Africa which together recorded 39 disease outbreaks and other acute public health events between 1 January and 30 October 2022.

**Figure:**
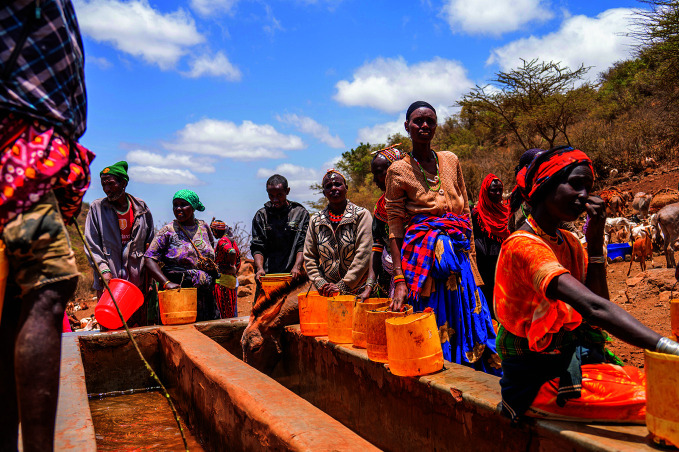
WHO/Billy Miaron.

## Uganda’s spreading Sudan virus outbreak

An outbreak of Sudan virus spread in Uganda. As of 7 November, 157 infections had been reported in eight districts (136 confirmed, 21 probable) and 53 people had been confirmed to have died of the disease. A further 21 people were assessed to have probably died of the same cause.

On 1 November the World Health Organization (WHO) raised its assessment of the risk posed by the outbreak from high to very high at the national level, and from low to high at the regional level.

The new assessment reflected a combination of factors, including the lack of licensed medical countermeasures to the virus, and its spread to urban areas, including Kampala which has a population of more than four million people and multiple connections to neighbouring countries.

There was also concern regarding reports of some high-risk contacts and symptomatic cases travelling between districts using public transportation, and the number of patients entering health facilities with suboptimal infection prevention and control practices.

While Uganda’s capacity to respond to Ebola outbreaks has increased in recent years, the health system may be challenged if the number of cases continues to rise and the outbreak spreads to other densely populated districts.

https://bit.ly/3WWsw7x

## Sudan virus vaccine trial

In related news, global health agencies outlined a plan to accelerate research on a vaccine for Sudan virus. Announced on 3 November, the plan will seek to build on lessons learned in responding to Zaire ebolavirus (another Ebola virus species) outbreaks.

There are currently no licensed vaccines for Sudan virus disease, but several candidates are to be tested in a clinical trial that will take place at Makerere University Lung Institute in Kampala.

A broad coalition of vaccine developers and funders are making the candidate vaccines available with the support of WHO, the Coalition for Epidemic Preparedness Innovations and Gavi, the Vaccine alliance.

http://bit.ly/3UMnMiJ

## Responding to the Horn of Africa crisis

The number of reported disease outbreaks and climate-related health emergencies in the Horn of Africa reached their highest level this century. This is according to a new assessment released by WHO on 3 November.

Most parts of the region are battling the worst drought in at least 40 years, with an unprecedented fifth rainy season failure now being predicted. Other areas face flooding and conflict, deepening a health crisis in which 47 million people are already facing acute hunger.

As of 3 November, WHO had made available over US$ 7 million in supplies and equipment, including kits for severe malnutrition, and had trained thousands of health workers in the management of the condition.

WHO has called for US$ 124 million to fund the emergency response, but as of 3 November only 34% of the funds required had been disbursed. Egmond Evers, acting incident manager for the WHO response, called for partners to come together to support urgently needed interventions. “We cannot delay any longer. We must act now,” he said.

https://bit.ly/3TabUWC

## Cholera in Lebanon

Lebanon is struggling to cope with its first cholera outbreak in over 30 years, reflecting a deterioration in the country’s economic situation and reduced access to clean water and proper sanitation services.

The outbreak was reported to WHO by the health ministry on 6 October. As of 7 November, 2722 people were reported to have been infected, 448 of those infections being laboratory-confirmed. Some 18 cholera-associated deaths had been reported.

WHO supported the ministry of health in securing a shipment of 600 000 doses of cholera vaccine from the International Coordination Group. The vaccines were delivered to a health ministry warehouse for distribution in a vaccination campaign designed to reach 70% of the target population.

https://bit.ly/3UuHIa2

## Health at COP27

WHO, in collaboration with the Wellcome Trust and partners, hosted the COP27 Health Pavilion at the COP27 UN Climate Conference which took place in Sharm El-Sheikh, Egypt from 6 to 18 November.

The pavilion hosted a range of global health stakeholders to ensure that health and equity were placed at the centre of the climate negotiations, showcasing evidence, initiatives and solutions to maximize the health benefits of tackling climate change.

On the eve of the conference, WHO issued a reminder of the impact that the climate crisis is having on public health and underlined the importance of putting considerations of health at the core of the COP27 negotiations.

https://bit.ly/3t6GRR6

https://bit.ly/3U50t3P

## Climate and health platform launched

The first global knowledge platform dedicated to climate and health was launched on 31 October by the Joint Office of WHO and the World Meteorological Organization, with support from the Wellcome Trust. The platform is a response to growing calls for actionable information needed to protect people from the health risks of climate change and other environmental hazards.

Bringing together the expertise and science of both organizations, the platform will serve as the go-to technical reference point for users of interdisciplinary health, environmental and climate science.

https://bit.ly/3FCUcrK

## New ACT-Accelerator plan

The Access to COVID-19 Tools (ACT) Accelerator, a global collaboration committed to accelerating the development, production and distribution of coronavirus disease 2019 (COVID-19) medical products, launched a new six-month plan, prioritizing long-term COVID-19 control.

Announced on 28 October, the plan outlines changes to the ACT-Accelerator’s set-up and activities to ensure that countries continue to be able to access COVID-19 medical products, while maintaining the collaboration’s capacity to help address future disease surges.

Specifically, the new plan focuses on research and development and market shaping to ensure a pipeline for new and enhanced COVID-19 medical products and securing institutional arrangements that will support sustained access for all countries to COVID-19 vaccines, tests and treatments, including oxygen. The ACT-Accelerator’s in-country work will focus on new product introduction and supporting the protection of priority populations.

https://bit.ly/3FH0lTY

**Cover photo**

A community health volunteer in Nepal counsels a pregnant woman regarding the importance of prenatal check-ups, diet and folic acid intake.

**Figure:**
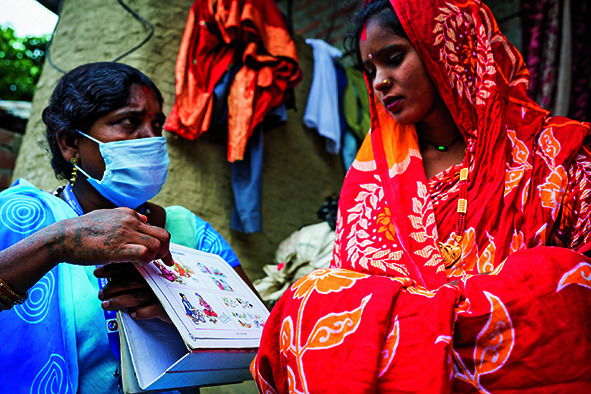
UNICEF/UN0638958/Upadhayay

## Vaccine access disparities

Poorer countries consistently struggle to access vaccines that are in-demand in wealthier countries. This is one of the key conclusions of the *Global vaccine market report 2022*, published by WHO on 9 November.

Limited vaccine supply and unequal distribution are among the drivers of global disparities in access; affordability is another. While prices tend to be tiered according to recipient country income, the report reveals that middle-income countries can pay as much – or even more – than wealthier ones for several vaccine products.

The report recommends focusing research efforts on WHO priority pathogens, ensuring transparency, facilitating technology transfer, and committing to specific equity-driven allocation measures.

http://bit.ly/3A2eRBZ

## New fungal pathogens list

WHO published a report presenting for the first time a list of fungal priority pathogens. The aim of the list is to focus and drive further research and policy interventions to strengthen the global response to fungal infections and antifungal resistance.

Published on 25 October, the WHO fungal priority pathogens list (WHO FPPL) comprises 19 fungi and is the first global effort to systematically prioritize fungal pathogens, taking into account their assessed public health importance and unmet research and development needs.

Fungal pathogens are becoming increasingly common and resistant to treatment with only four classes of antifungal medicines currently available, and few candidates in the clinical pipeline. Most fungal pathogens lack rapid and sensitive diagnostics and those that exist are not widely available or affordable globally.

https://bit.ly/3UGkz46

**Looking ahead**

5–7 December, 27th annual meeting of the Regions for Health Network. Brussels, Belgium. https://bit.ly/3TYcUOR

12 December, International Universal Health Coverage Day. http://bit.ly/3TtNs2N

16-17 January, 2023, International Conference on Public Health in the Digital Age. Zurich, Switzerland. http://bit.ly/3O8OY9h

